# Expanding Greenland seagrass meadows contribute new sediment carbon sinks

**DOI:** 10.1038/s41598-018-32249-w

**Published:** 2018-09-19

**Authors:** Núria Marbà, Dorte Krause-Jensen, Pere Masqué, Carlos M. Duarte

**Affiliations:** 1 Global Change Research Group, IMEDEA (CSIC-UIB), Institut Mediterrani d’Estudis Avançats, Miquel Marquès 21, 07190 Esporles (Illes Balears), Spain; 2 Department of Bioscience, Aarhus University, Vejlsøvej 25, DK-8600 Silkeborg, Denmark; 3 Arctic Research Centre, Aarhus University, Ny Munkegade 114, Building 1540, DK-8000 Aarhus C, Denmark; 4 Centre for Marine Ecosystems Research, School of Science, Edith Cowan University, 270 Joondalup Drive, Joondalup, WA 6027 Australia; 5 Departament de Física & Institut de Ciència i Tecnologia Ambientals, Universitat Autònoma de Barcelona, 08193 Bellaterra, Spain; 6 Oceans Institute & School of Physics, The University of Western Australia, 35 Stirling Highway, Crawley, WA 6009 Australia; 7 King Abdullah University of Science and Technology (KAUST), Red Sea Research Center (RSRC), Thuwal, 23955-6900 Saudi Arabia

## Abstract

The loss of natural carbon sinks, such as seagrass meadows, contributes to grenhouse gas emissions and, thus, global warming. Whereas seagrass meadows are declining in temperate and tropical regions, they are expected to expand into the Arctic with future warming. Using paleoreconstruction of carbon burial and sources of organic carbon to shallow coastal sediments of three Greenland seagrass (*Zostera marina*) meadows of contrasting density and age, we test the hypothesis that Arctic seagrass meadows are expanding along with the associated sediment carbon sinks. We show that sediments accreted before 1900 were highly ^13^C depleted, indicative of low inputs of seagrass carbon, whereas from 1940’s to present carbon burial rates increased greatly and sediment carbon stocks were largely enriched with seagrass material. Currently, the increase of seagrass carbon inputs to sediments of lush and dense meadows (Kapisillit and Ameralik) was 2.6 fold larger than that of sparse meadows with low biomass (Kobbefjord). Our results demonstrate an increasing important role of Arctic seagrass meadows in supporting sediment carbon sinks, likely to be enhanced with future Arctic warming.

## Introduction

The loss of natural carbon sinks through land use changes has contributed 32% of the cumulative anthropogenic CO_2_ emissions since the Industrial Revolution^1^, supporting a significant fraction of the resulting planetary warming^2^. Warming, in turn, is leading to further losses of some natural carbon sinks, such as seagrass meadows in the Mediterranean (*Posidonia oceanica*)^3^ and in Western Australia^4^, which rank amongst the most intense carbon sinks in the biosphere^5,6^.

However, global warming is also leading to a poleward migration of the leading edge of the biogeographical distribution of many terrestrial^7^ and marine species^8^. This trend has led to the hypothesis that marine macrophytes, including kelps and seagrass, would likely expand into the Arctic with future warming and reduced ice cover^9^. Whereas kelps already extend to very high latitudes (e.g. 80°N in Svalbard)^10^, the northern limit of eelgrass (*Zostera marina*), the seagrass species growing at highest latitudes in the Arctic^11^, is set around 70°N in areas influenced by warm Atlantic waters (e.g. in the Arctic coasts of Norway)^12^ and further south, around 64°N in Iceland^13^ and Western Greenland^14^. Hence, seagrass meadows are expected to expand into the Arctic with global warming^9,14^ and to develop new carbon sinks in the region. However, lack of observational time series preclude verification of these predictions.

Here we use paleoreconstruction of sediment accretion rates and sources of organic carbon to shallow coastal sediments to test the hypotheses that (a) seagrass meadows are expanding in the Arctic, and (b) that their expansion is enhancing carbon sinks in Arctic coastal sediments. We reconstruct the contribution of eelgrass to organic carbon burial in sediments under three contrasting eelgrass meadows in Western Greenland.

## Results and Discussion

**Figure 1:**
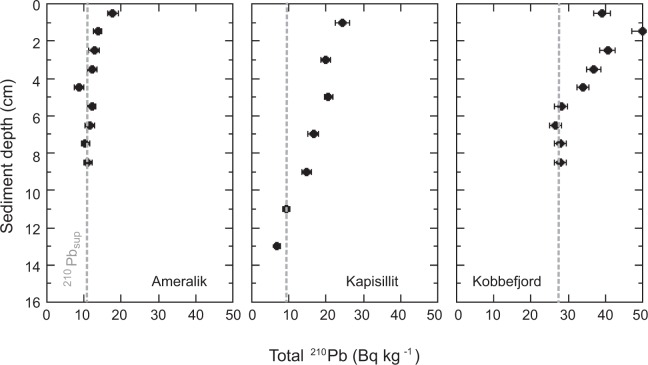
Sediment profiles of the total ^210^Pb concentrations in Ameralik, Kapisillit and Kobbefjord *Zostera marina* meadows. The dashed grey line indicates the supported ^210^Pb concentration (^210^Pb_sup_) at each meadow.

**Figure 2:**
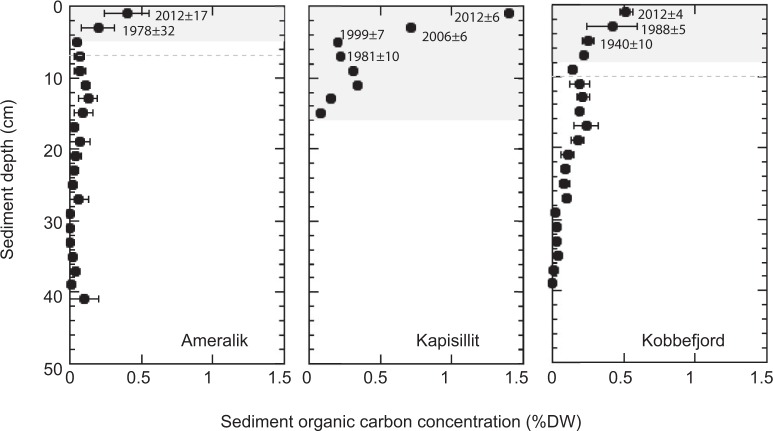
Sediment profile of organic carbon concentration in Ameralik, Kapisillit and Kobbefjord *Zostera marina* meadows. Grey background indicates the sediment horizon accreted since 1900, and the dashed grey horizontal line the associated standard error, considering the average sediment accretion rates obtained from the ^210^Pb age models, except for Kapisillit where the oldest sediment collected was 77 years old. The year when sediment was deposited on the top of each 2 cm sediment horizons dated with ^210^Pb CRS model is indicated.

The stocks of organic carbon (C_org_) within the top 10 cm of the sediments ranged from 197 g C m^−2^ to 595 g C m^−2^, and the inorganic carbon (C_inorg_) stocks were similar (Ameralik) or about 2 times lower (Kapisillit and Kobbefjord) than those of C_org_ (Table 1). The sediments under the seagrass meadows presented ^210^Pb concentration profiles that allowed establishing robust age models (Fig. 1), despite mixing may have occurred in the upper 2 cm sediment at Kobbefjord. The presence of a relatively thin mixed layer has limited impact when applying the CRS and the CF:CS (below the mixed layer) models^15^. Coupling of sediment chronologies with organic carbon concentration revealed recent increases in the C_org_ concentration of eelgrass sediments, particularly so for the dense seagrass meadow at Kapisillit over the past 40 years (Fig. 2).

**Table 1:** Mean (avg) and standard error (SE) of sediment organic and inorganic carbon stocks (within the top 10 cm sediment and in sediment accreted since 1900), organic and inorganic carbon burial rates and sediment accretion rate in Greenland eelgrass meadows.

	Ameralik		Kapisillit		Kobbefjord	
	avg	SE	avg	SE	avg	SE
C_org_ stock top 10 cm (g C m^−2^)	197	58	595		446	69
C_inorg_ stock top 10 cm (g C m^−2^)	175	31	358		240	13
C_org_ stock accreted after 1900 (g C m^−2^)	146	61	> 811*		404	90
C_inorg_ stock accreted after 1900 (g C m^−2^)	98	25	> 1129*		196	13
C_org_ burial rate after 1900 (g C m^−2^ yr^−1^)	1.30	0.55	10.53		3.60	0.81
C_inorg_ burial rate after 1900 (g C m^−2^ yr^−1^)	0.88	0.23	14.66		1.75	0.12
Sediment accretion rate (cm yr^−1^)	0.042	0.025	0.21	0.05	0.071	0.015
Aboveground biomass (g DW m^−2^)	330		300		90	
Belowground biomass (g DW m^−2^)	240		210		105	

*Zostera marina* biomass from Olesen *et al*.^14^.

^*^Kapisillit sediment core was only 16 cm long, which, given the estimated sediment accretion rate correspond to the accumulation of 77 years. Thus, the carbon stocks and burial rates at Kapisillit were estimated from 1935 onwards.

**Figure 3:**
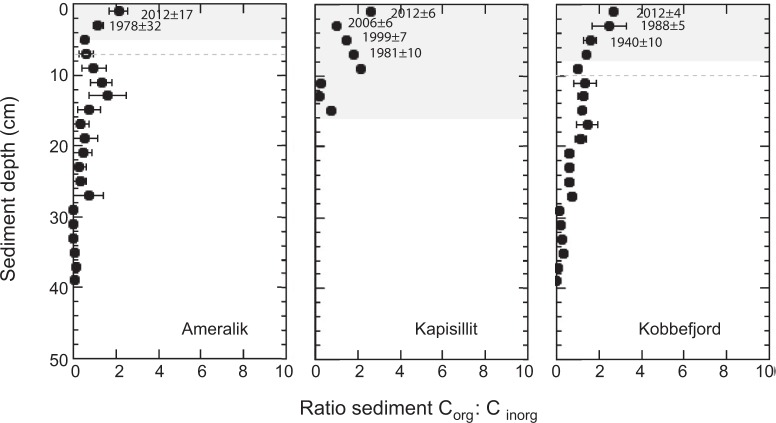
Sediment profile of organic carbon: inorganic carbon ratio in Ameralik, Kapisillit and Kobbefjord *Zostera marina* meadows. Grey background indicates the sediment horizon accreted since 1900, and the dashed grey horizontal line the associated standard error, considering average sediment accretion rates in Table 1, except for Kapisillit where the oldest sediment collected was 77 years old. The average year when sediment was deposited on the top of each 2 cm sediment horizons dated with ^210^Pb CRS model is indicated.

**Figure 4:**
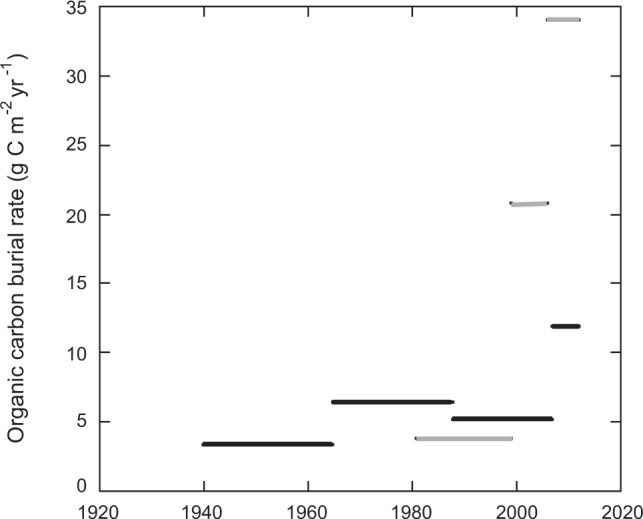
Organic carbon burial rates since 1940 in Kapisillit (grey) and Kobbefjord (black) *Zostera marina* meadows determined in the dated cores (one per site). Lines indicate the time period over which the sedimentation rate was estimated using the CRS ^210^Pb dating model. Data for Ameralik is not shown because the sediment dating model could only date two recent layers and, hence just one provide the rate of carbon accumulation over one time period.

The corresponding C_org_ burial rates since 1900 ranged 10 fold from 1.30 g C m^−2^ year^−1^ for that at Ameralik to 10.53 g C m^−2^ year^−1^ for the meadow at Kapisillit, with a large increase in the ratio of C_org_ to C_inorg_ towards present in all three meadows (Fig. 3), likely reflecting changes in sedimentary supply over time as well as differential diagenesis with sediment depth/age. Burial rates of C_org_ increased greatly from 1940’s to present in the two meadows (3.5 fold at Kobbefjord; 9.1 fold at Kapisillit) where rates could be resolved over this time period (Fig. 4). For Ameralik, only a few layers could be dated (since 1940) and, hence, we cannot resolve any potential change in carbon burial rates. Hence, the evidence for increased sedimentation rates toward present time is based on limited data and should be, therefore, considered to carry considerable uncertainty.

**Figure 5:**
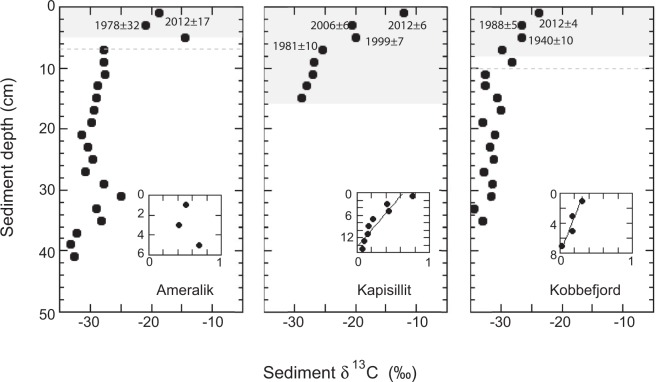
Sediment profile of δ^13^C_org_ (large plots) and the fraction of sediment organic carbon of seagrass origin (small plots, only for sediment horizon accreted after 1900) in Ameralik, Kapisillit and Kobbefjord *Zostera marina* meadows. Grey background in large plots indicates the sediment horizon accreted since 1900, and the dashed grey horizontal line represents the associated standard error, considering average sediment accretion rates in Table 1, except for Kapisillit where the oldest sediment collected was 77 years old. The average (±standard error) year when sediment was deposited on the top of each 2 cm sediment horizons dated with ^210^Pb CRS model is indicated. The continuous black line in small plots shows the linear regression equation fit to observations (Kapisillit: fraction C_seagr_ = −0.04 (±0.01) * sediment depth + 0.64 (±0.01), R^2^ = 0.82, N = 8, p < 0.005; Kobbefjord: fraction C_seagr_ = −0.04 (±0.01) * sediment depth + 0.33 (±0.04) R^2^ = 0.90, N = 4, p = 0.052).

Analysis of changes in Corg with sediment depth suggested a recent change in sources of C_org_ to the Greenland sediments examined. In particular, sediments accumulated before 1900 were characterized by highly ^13^C depleted C_org_ pools (mean ± SE = −30.44 ± 0.38‰ across all three sites), with C_org_ pools subsequently becoming progressively ^13^C-enriched (Fig. 5). The C_org_ source dominating sedimentary inputs before 1900 could be a combination of phytoplankton (δ^13^C = −24.7 ± 1.12‰) with land-derived C_org_, both from terrestrial vegetation and fossil organic carbon released with glacial melting, which have similar isotopic composition (δ^13^C extending to −35‰ in both cases)^16^. However, these sources cannot account for the ^13^C-enriched C_org_ pools stored in the sediment since 1900 (Fig. 5), which requires either a novel source more enriched in^13^C or an increase in the contribution of an existing source enriched in ^13^C. This is likely to be eelgrass-produced carbon, as the average (±SE) δ^13^C of present-day eelgrass is −7.24 ± 0.21‰, indicative of highly ^13^C-enriched organic carbon. The mixing model using the carbon sources before 1900, i.e. *business as usual carbon source scenario*, and eelgrass carbon as end members indicated an increased contribution of eelgrass to sediment C_org_ after 1900 in all meadows (Fig. 5). Indeed, the surface sediments in the eelgrass meadow at Kapisillit contain the largest contribution of eelgrass (see small plots in Fig. 5), consistent with the highest eelgrass biomass of these meadows, whereas the sediments at Kobbefjord have the lowest contribution of eelgrass to sediment C_org_, consistent with the low eelgrass biomass of Kobbefjord (Table 1)^14^. Moreover, the contribution of eelgrass material to sediment C_org_ pool has been increasing since 1900 at Kapisillit and Kobbefjord (see small plots in Fig. 5). It would be unlikely that the depletion of ^13^C observed in older sediment would result from diagenesis, since decomposition rate of eelgrass is about 8 times slower than that of phytoplankton^17^. The C_org_ stocks over the top 10 cm of sediment, where isotope mixing models unambiguously support the contribution of eelgrass, are low when compared to global seagrass C_org_ stocks within a similar soil thickness (2.0 to 6.0 Mg C_org_ ha^−1^ in the meadows studied here compared to 9.6 ± 0.7 Mg C_org_ ha^−1^, on average ± SE, recalculated from the global compilation of Fourqurean *et al*.^6^).

Warming and reduced ice cover in Greenland fjords have been proposed to be conducive to a poleward expansion of marine macrophytes^9,14,18,19^, as both light and temperature thresholds become more favourable to support macroalgal and seagrass growth. Indeed, experimental evidence indicates that Greenland current and projected (under IPCC scenarios of greenhouse gas emissions) warming conditions enhance eelgrass growth^20^. The presence of eelgrass in Greenland fjords was first documented in the Godthåbsfjord system in 1830^14^, and in Kapisillit and Ameralik in 1916 (Herbarium specimens from Greenland Herbarium, Botanical Museum, University of Copenhagen) and 1921^21^. However, eelgrass in Kobbefjord, the population with the smallest extent and biomass among those studied here, wasn’t reported until 2009^14^. Hence, we speculate that while eelgrass meadows have been present in Greenland for at least 180 years, they appear to be expanding and increasing their productivity. This is supported by the rapid growth in the contribution of seagrass-derived carbon to the sediment C_org_ pool, from less than 7.5% at the beginning of 1900 to 53% at present, observed in the studied meadows. Expansion and enhanced productivity of eelgrass meadows in the subarctic Greenland fjords examined here is also consistent with the on average 6.4-fold acceleration of C_org_ burial in sediments between 1940 and present.

Seagrass meadows have been shown to rank amongst the most intense carbon-sink ecosystems of the biosphere^6,22,23^ with conservation and restoration programs aimed at protecting and restoring the carbon stocks and sink capacity lost with global seagrass decline^24,25^. In contrast, seagrass meadows in Greenland seem to be expanding, propelled by warmer seawater temperature and higher doses of submarine irradiance with reduced ice cover^14^. The expansion of seagrass in Greenland fjords represents a novel carbon sink, with limited significance at present due to the small size of the meadows. However, the potential for further expansion is huge, as the convoluted Greenland coastline represents about 12% of the global coastline. The poleward latitudinal limit of eelgrass is located at far higher latitudes (70°N) than those studied here (64°N), suggesting that eelgrass, along with other boreal macrophyte species, is likely to expand poleward with decreasing ice cover and higher temperatures^9,14,26^. Hence, whereas the carbon sink associated with sediments under Greenland eelgrass meadows is likely to be very modest at present, it may reach significant levels along the 21^st^ century.

Whereas the concern globally is in slowing down or stopping altogether further losses of seagrass^24,27^, we provide evidence here for an increasingly important role of sediments under seagrass meadows in Greenland as a carbon sink, whose significance is likely to increase with further climate warming. Propelling this emerging carbon sink requires protection of extant meadows, as these do not only represent carbon sinks already in operation, but also supply propagules essential for further expansion of this valuable ecosystem.

## Methods

### Study sites

The study was conducted in 3 subarctic seagrass (*Zostera marina*) meadows located in 3 fjords of the Godthåbsfjordsystem, near Nuuk (West Greenland), the region that harbours all but one *Z*. *marina* meadows reported so far in West Greenland^14^. The fjords are 17 km to 100 km long, with the inner parts of the branches covered with sea ice from November to May during severe winters and no sea ice in the outer bays^14^. The tidal amplitude in the region ranges from 2 to 5 m.

Two of the studied seagrass meadows (Ameralik, 64°15′N, 51°35′W; and Kapisillit, 64°28′N, 50°13′W) were located at the inner branches of the Ameralik-Itivleq and Kapisigdlit Kangerdluat fjords whereas the third meadow grew at the middle part of Kobbefjord (64°09′N, 51°33′W). All studied seagrass meadows were permanently submerged, at water depths ranging between 2 and 4 m, and exposed to maximum summer water temperatures of 9 °C at Kobbefjord, to 14–15 °C, at Ameralik and Kapisillit^14^. The presence of *Z*. *marina* meadows at Kapisillit and Ameralik were already documented in 1916 (observer O. Bendixen, herbarium collection, Botanical Museum, Copenhagen, Denmark) and 1921^21^, while the first observations of seagrass at Kobbefjord, a thoroughly investigated area (228 documents including the keyword “Kobbefjord” in citations in Google Scholar, 20 June 2017) date from 2009. The absence of pre-2009 Kobbefjord meadow historical accounts strongly suggests that this meadow is relatively young^14^. *Z*. *marina* at Ameralik and Kapisillit formed lush and extensive meadows, with 100% cover and high above- and belowground biomass (Table 1). Conversely, *Z*. *marina* at Kobbefjord developed only few patches of vegetation, with less than 10% cover and lower total summer biomass (Table 1). Olesen *et al*.^14^ provide a detailed description of *Z*. *marina* dynamics and environmental conditions at the studied sites.

### Sampling

In August 2012, we collected sediment cores (5.2 cm diameter) in seagrass meadows of the 3 study sites. We retrieved 3 cores from Kobbefjord and 4 cores from Ameralik, each being 36–42 cm long, and a single 16 cm long core from the meadow at Kapisillit. The sediment at Kapisilit was extremely rich in clay and it prevented to insert the cores deeper; despite trying we could only collect one sediment core longer than 10 cm. We collected the sediment cores from a boat using a manual sampling device to insert the corer as deep as possible into the sediment. Because of the method to collect the cores, we could not measure sediment compaction due to sampling, but it should be negligible, at least, for Kobbefjord and Ameralik sediments, since they were highly sandy and organic poor. The seagrass meadows sampled extended along the suitable habitat. However, we did not collect samples in bare sediments adjacent to vegetated ones, because their current bare nature may be transient and there is no guarantee that these sediments would not have supported seagrass at some point along the 100 years of sediment carbon burial reconstructed here.

We transported the sediment cores vertically to the Greenland Institute of Natural Resources, Nuuk. In the laboratory, we sliced one core from Ameralik and Kobbefjord every centimeter and the remaining cores every 2 cm. We weighed all sediment samples after oven-drying them at 60 °C for 48 h, and estimated their sediment dry bulk density by dividing sediment dry weight by wet sediment volume. We ground the dried sediment and stored the samples until analysis of organic matter, inorganic carbon concentrations, δ^13^C and ^210^Pb concentrations.

Along with the collection of sediment cores we also collected 3 samples of eelgrass leaves, each containing material from different shoots, from each meadow, dried at 60 °C for 48 h and ground them for subsequent analysis of δ^13^C (see below).

### ^210^Pb sediment dating and sediment accretion rates

The cores were dated by means of ^210^Pb. Concentrations of ^210^Pb along the upper 10–20 cm of one core at each site were determined by alpha spectrometry through the measurement of the activity of its granddaughter, ^210^Po, following Sanchez-Cabeza *et al*.^28^. Briefly, 200 mg aliquots of each layer were spiked with a known amount of ^209^Po, acid digested and dissolved into a 100 mL 1 M HCl solution, from which the polonium isotopes were autoplated onto pure silver disks. Polonium emissions were measured by alpha spectrometry using Passivated Implanted Planar Silicon, PIPS detectors (CANBERRA, Mod. PD-450.18 A.M.). Reagent blanks were run in parallel and found to be comparable to the detector backgrounds. Supported ^210^Pb concentrations were determined by averaging total ^210^Pb concentrations at the base of each profile. These were comparable to the ^226^Ra concentrations obtained at selected depths in each core by measuring the emissions of its decay products ^214^Pb (295 and 352 keV peaks) and ^214^Bi (609 keV peak) using a high-purity germanium detector (CANBERRA, mod. GCW3523) in calibrated geometries, sealed for 21 days.

The concentration profiles of excess ^210^Pb used for the age modelling were determined by subtraction of the supported ^210^Pb from total ^210^Pb concentrations along each core (Fig. 1), showing a decrease in concentration from the surface to constant concentrations at various depths depending on each core (excess ^210^Pb horizon). Sediment accumulation rates were calculated by applying the constant flux: constant sedimentation (CF:CS)^29^ and the constant rate of supply (CRS)^30^ models, which rendered equivalent results (Table 1).

### Organic and inorganic carbon stocks, burial rates and δ^13^C

We measured the concentration of organic matter (OM, % DW) using the loss of ignition technique along the sediment depth profile at 2 cm resolution, and in subsamples of 3 sediment cores per meadow, except for Kapisillit where only one core was collected. We combusted pre-weighed dry (60 °C) sediment samples at 550 °C for 5 hours and estimated the concentration of organic matter (OM) as:

$${\rm{OM}}=({{\rm{W}}}_{60}-{{\rm{W}}}_{550})\ast 100/{{\rm{W}}}_{60},$$

where W_60_ is the dry weight of the sample at 60 °C and W_550_ the weight of the sample after combustion at 550 °C. We estimated the sediment organic carbon concentrations (C_org_, %DW) from measured organic matter concentrations (OM, % DW) using the relationship described for seagrasses by Fourqurean *et al*.^6^.

We also analyzed the concentration of inorganic carbon (C_inorg_, %DW) by conducting a second combustion of the sediment samples at 1000 °C for 2 h to release the CO_2_ of the carbonate and subsequently calculating the concentration of C_inorg_ as:

$${{\rm{C}}}_{{\rm{inorg}}}=[({{\rm{W}}}_{550}-{{\rm{W}}}_{1000})\ast 100/{{\rm{W}}}_{60}]\ast 0.27,$$

where W_1000_ was the weight of the sediment sample after the second combustion and 0.27 is the ratio of the atomic weight of carbon (12 g) to the molecular weight of CO_2_ (44 g) released during carbonate combustion. Along the sediment profiles of each core, we calculated the ratio between organic and inorganic carbon concentrations (C_org_: C_inorg_) as well as the pools of C_org_ (g C_org_ cm^−3^) and C_inorg_ (g C_inorg_ cm^−3^) by multiplying, respectively, C_org_ and C_inorg_ concentrations with the sediment dry bulk density of each sediment sample.

We calculated the stocks of C_org_ and C_inorg_ (in g C m^−2^) within the top 10 cm *Z*. *marina* sediments by integrating the C_org_ and C_inorg_ pools within the top 10 cm sediment layer and over one meter square of seagrass meadow. Similarly, we calculated the C_org_ and C_inorg_ stocks (in g C m^−2^) accumulated since year 1900 by integrating, respectively, C_org_ and C_inorg_ pools in sediments younger than 112 years, using the sedimentation rates obtained from the ^210^Pb data.

We estimated average annual C_org_ and C_inorg_ burial rates (in g C m^−2^ yr^−1^) in the Greenland seagrass meadows since year 1900 by dividing, respectively, the C_org_ and C_inorg_ stocks accreted since then by 112 years (77 years for Kapisillit since the core was only 16 cm long and, hence, did not encompass an entire century of sedimentation). We also estimated the C_org_ and C_inorg_ burial rates over shorter time periods using the sediment age estimates along the sediment profile derived from the CRS ^210^Pb dating model. We calculated the carbon (C_org_ or C_inorg_) burial rates for a particular time period by dividing the carbon stock accumulated within that period by the length of the time period (in years).

We analyzed the δ^13^C-signature of the organic carbon in all sediment segments of one core from each seagrass meadow and in the leaves of the seagrass shoots from each Greenland *Z*. *marina* meadow. For the sediment samples, the isotopic analyses were conducted after acidifying the samples in order to remove calcium carbonate to avoid bias in the isotopic signature^31^. Acidification was conducted in a fume hood with saturated HCL for 24 h. The stable carbon isotopic composition was analysed by an isotope ratio mass spectrometer (Thermo fisher scientific). It is reported in the δ notation as the ratio of the ^13^C to the ^12^C isotope in the sample (Rsample) relative to that of a standard (Standard) i.e., δ sample = 1000 [(Rsample/Rstandard) − 1]. The primary standard is Vienna Pee Dee Bellemnite (VPDB) and secondary standards are Acetanilide (Schimmelmann) and sucrose.

We tested if the relative contribution (f) of seagrasses to sediment organic carbon increased after year 1900. We did so by applying a two source-mixing model,

$${{\rm{d}}}^{13}{{\rm{C}}}_{{\rm{sed}}{\rm{after}}1900}={{\rm{d}}}^{13}{{\rm{C}}}_{{\rm{seagr}}}\ast {\rm{f}}+[{{\rm{\delta }}}^{13}{{\rm{C}}}_{{\rm{sed}}{\rm{before}}1900}\ast (1-{\rm{f}})],$$

that considered *Z*. *marina* (δ^13^C_seagr_ Ameralik = −7.31 ± 0.02‰, δ^13^C_seagr_ Kapisillit = −6.58 ± 0.33‰, δ^13^C_seagr_ Kobbefjord = −7.83 ± 0.15‰) and a *business as usual carbon source scenario*, represented by the average δ^13^C_sed_ observed in sediments accreted before year 1900 (δ^13^C_sed after 1900_ = −30.44 ± 0.38‰), as end members. We corrected for the historical change in the δ^13^C source signatures due to ^13^C depletion in the atmospheric CO_2_ and oceanic DIC δ^13^C signature towards present derived from the burning of fossil fuels (i.e. Suess effect)^32^. This was done by applying the model described by Schelske and Hodell^33^ and modified by Verburg^34^:

$${{\rm{d}}}^{13}{{\rm{C}}}_{{\rm{atm}}}=4577.8-7.343\ast {\rm{Y}}+3.9213\ast {10}^{-3}\ast {{\rm{Y}}}^{2}-6.9812\ast {10}^{-7}\ast {{\rm{Y}}}^{3}$$

to estimate the δ^13^C of atmospheric CO_2_ (δ^13^C_atm_) over time (years, Y) since year 1840. These values were subsequently normalized to δ^13^C_atm_ in year 1840, and the resulting time-dependent depletion in δ^13^C since1840 was subtracted from the measured δ^13^C_sed_ for each dated sediment section.

### Data analysis

Average (and standard error) values of carbon parameters were calculated from measurements of 3 replicated sediment cores from Ameralik and Kobbefjord while values from a single core were applied from Kapisillit. We used JMP to fit linear trends to sediment profiles of enhanced eelgrass contribution (relative to “business as usual” organic carbon input sources, i.e. those prior 1840) to sediment organic carbon after year 1900.

## Data Availability

Data accessible at Digital CSIC URI: http://hdl.handle.net/10261/169555 ^35^.
